# Noninvasive intravital high-resolution imaging of pancreatic neuroendocrine tumours

**DOI:** 10.1038/s41598-019-51093-0

**Published:** 2019-10-10

**Authors:** Mirela Balan, Marta Trusohamn, Frank Chenfei Ning, Stefan Jacob, Kristian Pietras, Ulf Eriksson, Per-Olof Berggren, Daniel Nyqvist

**Affiliations:** 1 Division of Vascular Biology, Department of Medical Biochemistry and Biophysics, Karolinska Institutet, Stockholm, 17165 Sweden; 2 The Rolf Luft Research Center for Diabetes and Endocrinology, Karolinska Institutet, Karolinska University Hospital L1, Stockholm, 17165 Sweden; 3 Division of Translational Cancer Research, Department of Laboratory Medicine, Lund University, Lund, 22381 Sweden

**Keywords:** Cancer imaging, Cancer models, Tumour angiogenesis, Endocrine cancer

## Abstract

Preclinical trials of cancer drugs in animal models are important for drug development. The Rip1Tag2 (RT2) transgenic mouse, a model of pancreatic neuroendocrine tumours (PNET), has provided immense knowledge about PNET biology, although tumour progression occurs in a location inaccessible for real-time monitoring. To overcome this hurdle we have developed a novel platform for intravital 3D imaging of RT2 tumours to facilitate real-time studies of cancer progression. Pre-oncogenic islets retrieved from RT2 mice were implanted into the anterior chamber of the eye (ACE) of host mice, where they engrafted on the iris, recruited blood vessels and showed continuous growth. Noninvasive confocal and two-photon laser-scanning microscopy through the transparent cornea facilitated high-resolution imaging of tumour growth and angiogenesis. RT2 tumours in the ACE expanded up to 8-fold in size and shared hallmarks with tumours developing *in situ* in the pancreas. Genetically encoded fluorescent reporters enabled high-resolution imaging of stromal cells and tumour cell migration. Sunitinib treatment impaired RT2 tumour angiogenesis and growth, while overexpression of the vascular endothelial growth factor (VEGF)-B increased tumour angiogenesis though tumour growth was impaired. In conclusion, we present a novel platform for intravital high-resolution and 3D imaging of PNET biology and cancer drug assessment.

## Introduction

Preclinical trials of cancer drugs in animal models are an important step in the drug development process where genetically engineered mouse models represent a valuable platform that may inform about success or failure in humans^1,2^. The Rip1Tag2 (RT2) transgenic mouse model represents a widely used model of pancreatic neuroendocrine tumours (PNET)^3^. In this model, the simian virus 40 large T-antigen (Tag) oncogene is expressed under the control of the rat insulin gene promoter (Rip) leading to multifocal development of insulin-producing beta cell carcinoma (insulinoma) in the pancreatic islets^3^. Although all beta cells express the Tag oncogene at birth, stochastic events will add further genetic and epigenetic changes that are required for successful stepwise carcinogenesis^4^. The RT2 model has proven highly instrumental for preclinical validation of eligible biological or pharmacological anti-angiogenic compounds, some of which have been subsequently successfully implemented into clinical practice^2^. Recent, cross-species comparison between human PNETs and the RT2 model has also revealed that this mouse model shares many similarities with human PNETs^5^. Despite successful application of the RT2 model, current investigations are limited by the lack of techniques supporting temporal and high-resolution studies of PNET progression.

The application of imaging techniques in both medicine and research has proven to be crucial for the understanding of the structure and function of many tissues in health and disease^6^. Clinical imaging modalities, such as PET, MRI, CT, etc., allow a view of whole organs in a noninvasive fashion. However, the low spatial resolution of these modalities prevents visualization of the earliest stages of disease onset and determination of cause-and-effect relationships among cells during tumour progression^6^. Intravital microscopy by confocal and multiphoton imaging in combination with sophisticated genetic models and animal preparations (e.g., window models) can overcome these limitations and provide high-resolution visualization of cellular and sub-cellular structures^6,7^. Hence, intravital microscopy has successfully enabled detailed and dynamic investigations on the cellular level in multiple tumour preparations, including pancreatic cancer^6,8,9^. However, intravital imaging of internal organs presents several challenges that arise from both the inaccessibility and the constant motion of these organs. This applies to the pancreas and especially to the pancreatic islets, embedded microorgans associated with many pathologies, including cancer and diabetes.

We have previously developed a platform for intravital imaging of pancreatic islets transplanted to the anterior chamber of the eye (ACE), which facilitates noninvasive confocal and multiphoton imaging of pancreatic islet revascularization and beta cell function^10,11^. Here, we have developed this platform further by establishing a protocol for implantation of pre-oncogenic RT2 tumours to the ACE, and by developing a protocol for 3D vascular quantification. Hence we present a platform for noninvasive intravital 3D imaging of RT2 tumour progression. Pre-oncogenic islets where isolated from young RT2 mice prior to the onset of oncogenic growth and implanted into the ACE of recipient mice. The RT2 islets engrafted on the iris where they recruited blood vessels and commenced to expand. Noninvasive imaging facilitated repetitive high-resolution 3D visualization and quantification of tumour growth, by label free imaging of scattered light, and of tumour angiogenesis, by imaging of systemic administered fluorescent dextrans. The application of fluorescent reporter mice facilitated visualization of distinct cell populations within the tumour microenvironment, and imaging of tumour cell migration. Treatment with Sunitinib caused vessel regression and impaired expansion of RT2 tumours located in the ACE, validating functional assessment of drug efficacy. Intravital imaging of individual RT2 tumours overexpressing the vascular endothelial growth factor (VEGF)-B revealed that VEGF-B strongly repressed tumour growth, while tumour vascularization was increased. Taken together, we present a novel platform for noninvasive intravital imaging of RT2 tumour progression that provides 3D, high-resolution and dynamic assessment of PNET biology and cancer drug efficacy.

## Results

### Noninvasive intravital imaging of RT2 tumour growth and angiogenesis

**Figure 1:**
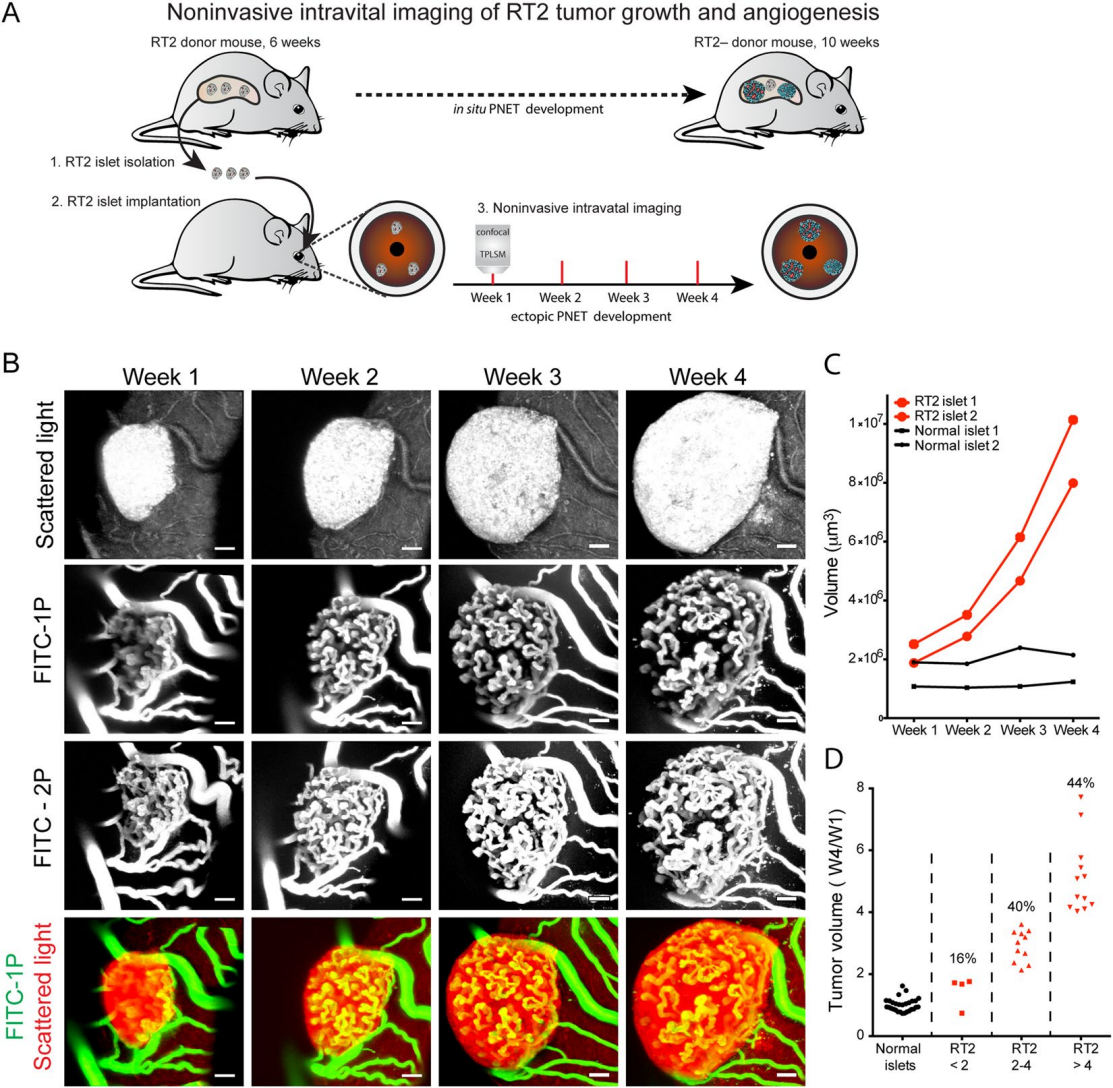
Noninvasive intravital imaging of RT2 tumour growth and angiogenesis. (**A**) Graphic illustration of the methodological approach; 1) Isolation of pre-oncogenic RT2 islets, 2) Implatation of pre-oncogenic islets into the anterior chamber of the eye (ACE), 3) Noninvasive intravital imaging of tumour progression. (**B**) Image panel (maximum projections) of a RT2 tumour imaged at indicated time-points following implantation. The tumour cells were imaged by detection of scattered light (grey scale), and the tumour vasculature were imaged by systemic administration of FITC-dextran 500 kDA captured by either confocal (1 P) or TPSLM imaging (2 P) (grey scale). Bottom row represents the merged scattered light (grey scale) and FITC-1P (green). (**C**) The graph displays the size of two normal pancreatic islets and two oncogenic RT2 islets during four weeks following implantation into the same ACE. (**D**) The graph shows variation in RT2 tumour growth four weeks after implantation by displaying the fold changes in growth comparing Week 4 to Week 1 (W4/W1) (RT2 tumours = 27, normal islets = 29). Scale bars represent 50 μm.

Non-hyperplastic pancreatic islets, hereafter termed “pre-oncogenic”, from 6-week-old RT2^+/−^ transgenic mice^3^ were isolated and cultured for two days, before implantation into the anterior chamber of the eye (ACE) of syngeneic recipients (Fig. 1A). The implanted RT2 islets engrafted on the iris and could be readily observed macroscopically through the transparent cornea (Supplement Fig. 1A). To compare and characterize the engraftment process of oncogenic RT2 islets, normal non-oncogenic (RT2^−/−^) pancreatic islets where also implanted into the same eye. Confocal microscopy facilitated imaging of scattered light, i.e. non-fluorescent light dispersed by the tissue^12,13^, and thereby enabled visualization of non-labelled tumour cells (Fig. 1B). Intravenous administration of FITC-dextran 500 kDa at the time of imaging facilitated visualization of blood vessels with both confocal and two-photon laser-scanning microscopy (TPLSM) imaging, where TPLSM allowed visualization of vessels at a greater depth in the islets compared to confocal microscopy (Fig. 1B).

Following implantation, normal islets maintained their original volume and became revascularized through angiogenic growth of iris vessels into the islets (Fig. 1C and Supplement Fig. 1B), as previously reported^10,11^. Within 2 to 4 weeks after transplantation the normal islets were vascularized with tortuous vessel networks formed by uniformed sized capillaries (Supplementary Figs 1B and 4), similar as previously described in detail^11,14^. In contrast to normal islets, oncogenic RT2 islets expanded in size following implantation and rapidly increased their volume (Fig. 1B,C). Oncogenic RT2 islets showed a strong angiogenic capacity and rapidly recruited a dense vessel network that continuously expanded during tumour progression (Fig. 1B). Similar to normal islets, the RT2 tumours acquired a tortuous vasculature but with capillaries that frequently displayed an irregular shaped and wider diameter (Fig. 1B). Real-time imaging of RT2 tumours displayed an irregular capillary blood flow, and revealed some capillaries with alternating flow directions (Supplement Movie 1).

The *in situ* tumour development in the pancreas of RT2 mice is characterized by a heterogeneous transformation of the islet population where 50% of the islets become hyperplastic at 5–7 weeks of age, 10% reaches an “angiogenic stage” by 7–12 weeks, and 2–4% becomes large tumours^15^. Interestingly, we observed a high degree of oncogenic transformation following implantation of pre-oncogenic RT2 islets into the ACE. Only a minority of the pre-oncogenic islets, 16%, did not expand and retained their original size, while 40% showed 2–4-fold volume increase, and 44% expanded 4–8-fold in volume, measured at 4 weeks following implantation (Fig. 1D).

### Spatial and temporal imaging of stromal and tumour cell populations

**Figure 2:**
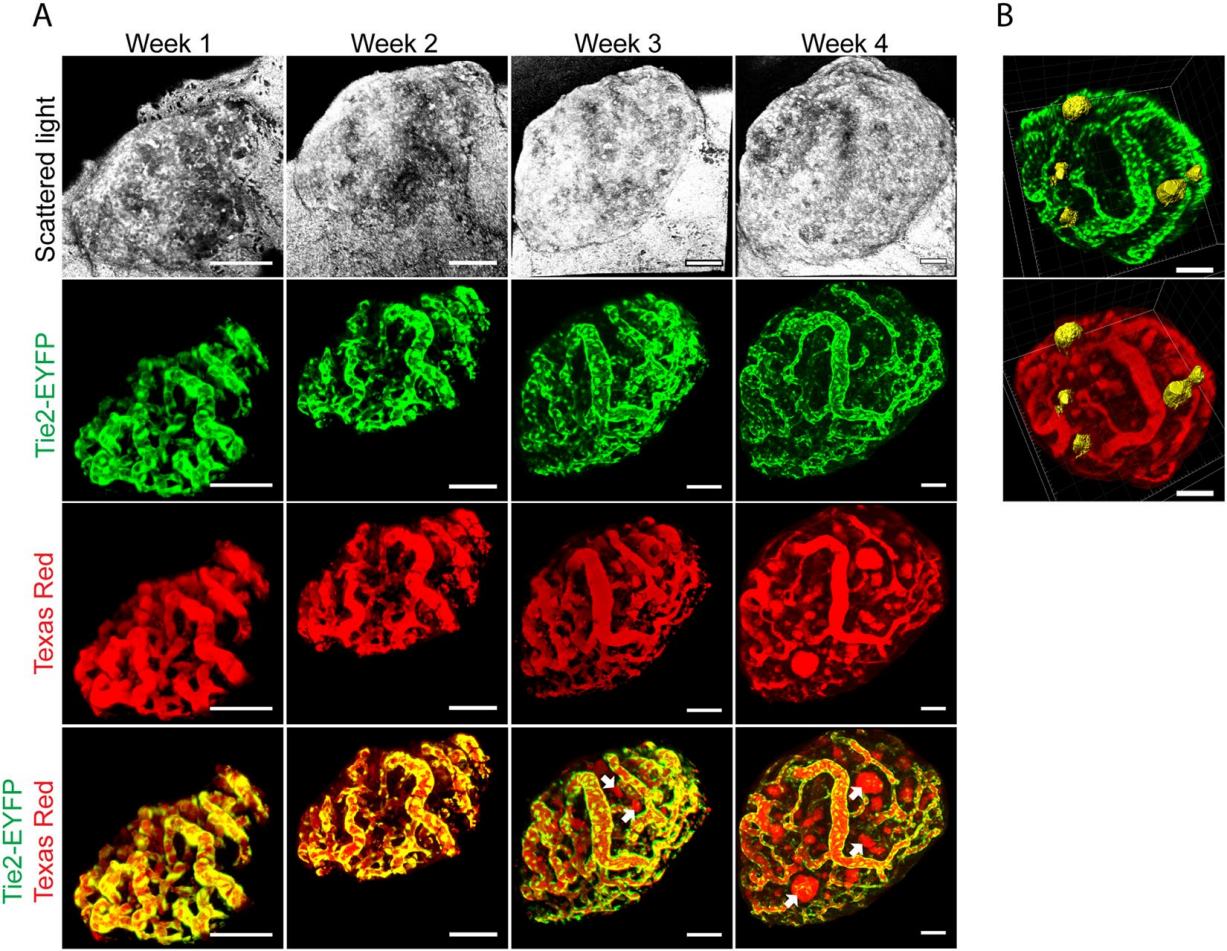
High-resolution of tumour stromal cells. (**A**) Image panel showing a RT2 tumour following implantation into a Tie2-EYFP host at indicated time-points. Tumour cells were visualized by scattered light (grey scale). Tumour vessels were visualized by EYFP expressed by endothelial cells (and through Texas Red-70 kDa dextran administered to the circulation (Texas Red) (red). Intratumour cavities, i.e. blood islands, filled by Texas Red dye but lacking endothelial lining were observed from week 3 (arrows). Bottom row represents merged images (n = 7). (**B**) The 3D image projections display the localization of intratumour cavities (yellow) in relation to the tumour vasculature. Scale bars represent 50 μm.

To enable monitoring of tumour angiogenesis during cancer progression, we implanted pre-oncogenic RT2 islets into Tie2-EYFP reporter mice, characterized by endothelial expression of the enhanced yellow fluorescent protein (EYFP), and obtained by crossing Tie2-Cre mice^16^ with R26R-EYFP(Ai3) mice^17^. Tumour blood vessels could be visualized in great detail by EYFP expressed by the endothelial cells, and repeated imaging facilitated 3D monitoring of the intratumoural vasculature network during tumour expansion (Fig. 2A). Administration of Texas Red 70 kDa to the circulation showed that all tumour vessels were functionally connected and perfused (Fig. 2A). After three weeks of tumour growth and onwards, Texas Red dye was observed to accumulate at distinct locations inside the tumours (Fig. 2A, arrows). 3D projections revealed that these dye filled spaces were not lined with endothelial cells and located apart from blood vessels, suggesting that they represent intratumoural cavities (Fig. 2B). These cavities are similar to the “blood islands”, intratumoural cavities filled with red blood cells that arise during *in situ* development of RT2 tumours^18,19^. The intratumoral cavities could also be visualized by imaging the scattered light of tumour cells (Supplement Movie 2). In addition to EYFP expressing endothelial cells, we could also observe round solitary EYFP expressing cells that were not associated with blood vessels. Most likely these cells represent Tie2 positive macrophages^20^.

**Figure 3:**
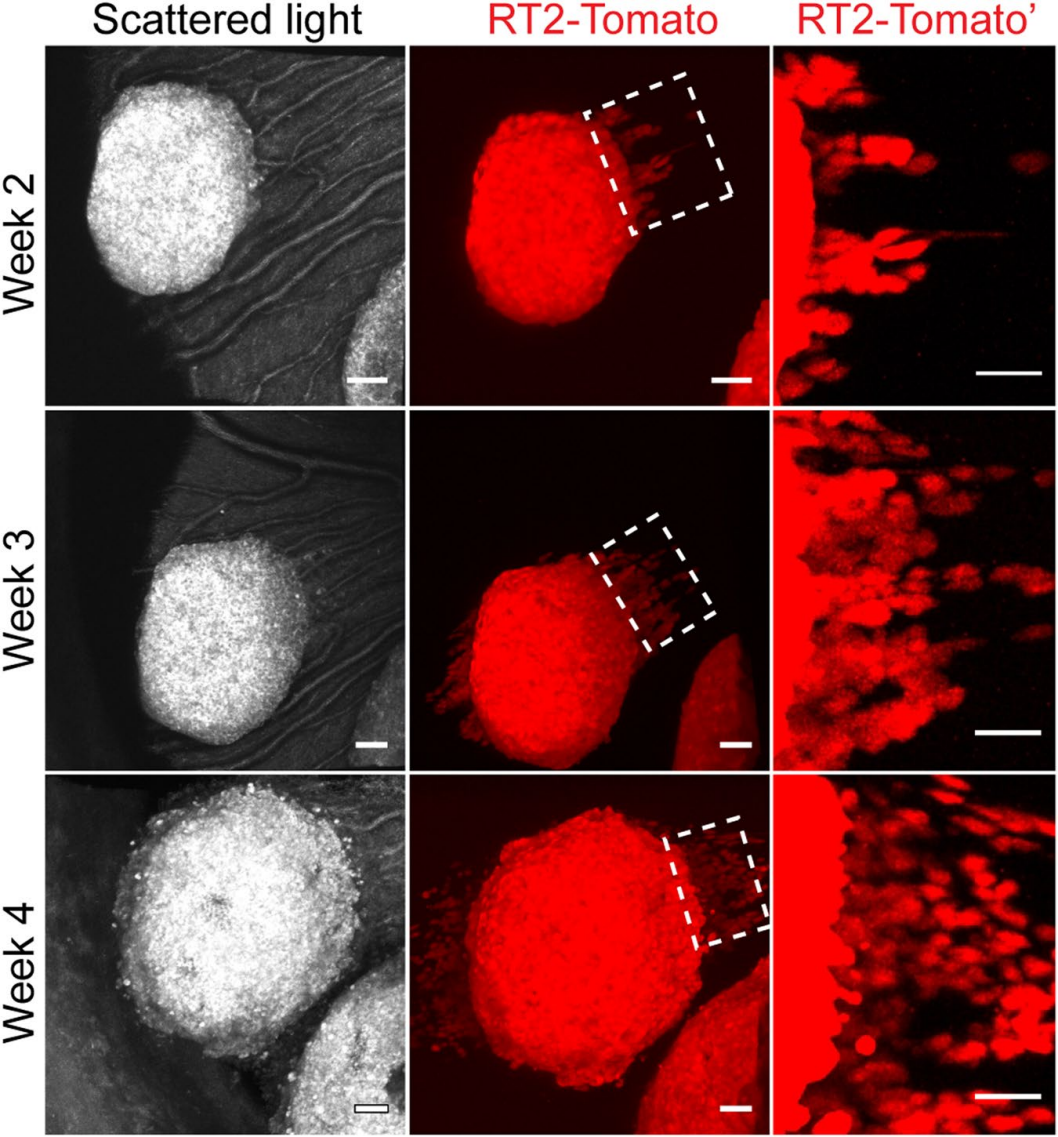
High-resolution imaging of tumour cell migration. (**A**) Image panel showing a RT2-Tomato tumour following implantation into the ACE at indicated time-points. Tumour cells were visualized by scattered light (grey scale) and tdTomato fluorescence (red). The right panel “RT-Tomato” represents magnifications of the indicated boxed areas (dashed line) and displays tumour cell migration. (n = 20). Scale bars: 50 μm mid panel, and 30 μm right panel.

To enable visualization of tumour cells at single cell resolution, RT2 transgenics were intercrossed with RIP-Cre^21^ and R26R-Tomato(Ai14)^17^ mice to generate RT2-Tomato mice, characterized by expression of the fluorescent tdTomato protein by tumour cells. Imaging of RT2-Tomato tumours in the ACE following implantation facilitated visualization of tumour cells at a greater detail and depth compared with imaging of scattered light (Fig. 3 and Supplement Movie 3). Following two weeks of tumour growth, single tumour cells could be observed to migrate away from the tumours along the iris, indicating a metastatic capacity (Fig. 3). Repetitive imaging allowed monitoring of cancer cell motility and revealed continuous migration of single cells (Fig. 3). Fixation and sectioning of RT2-Tomato tumours confirmed that RT2 tumours grew on top of the iris, and that tdTomato fluorescence correlated with insulin staining (Supplemental Fig. 2).

### 3D quantification of tumour growth and tumour angiogenesis

**Figure 4:**
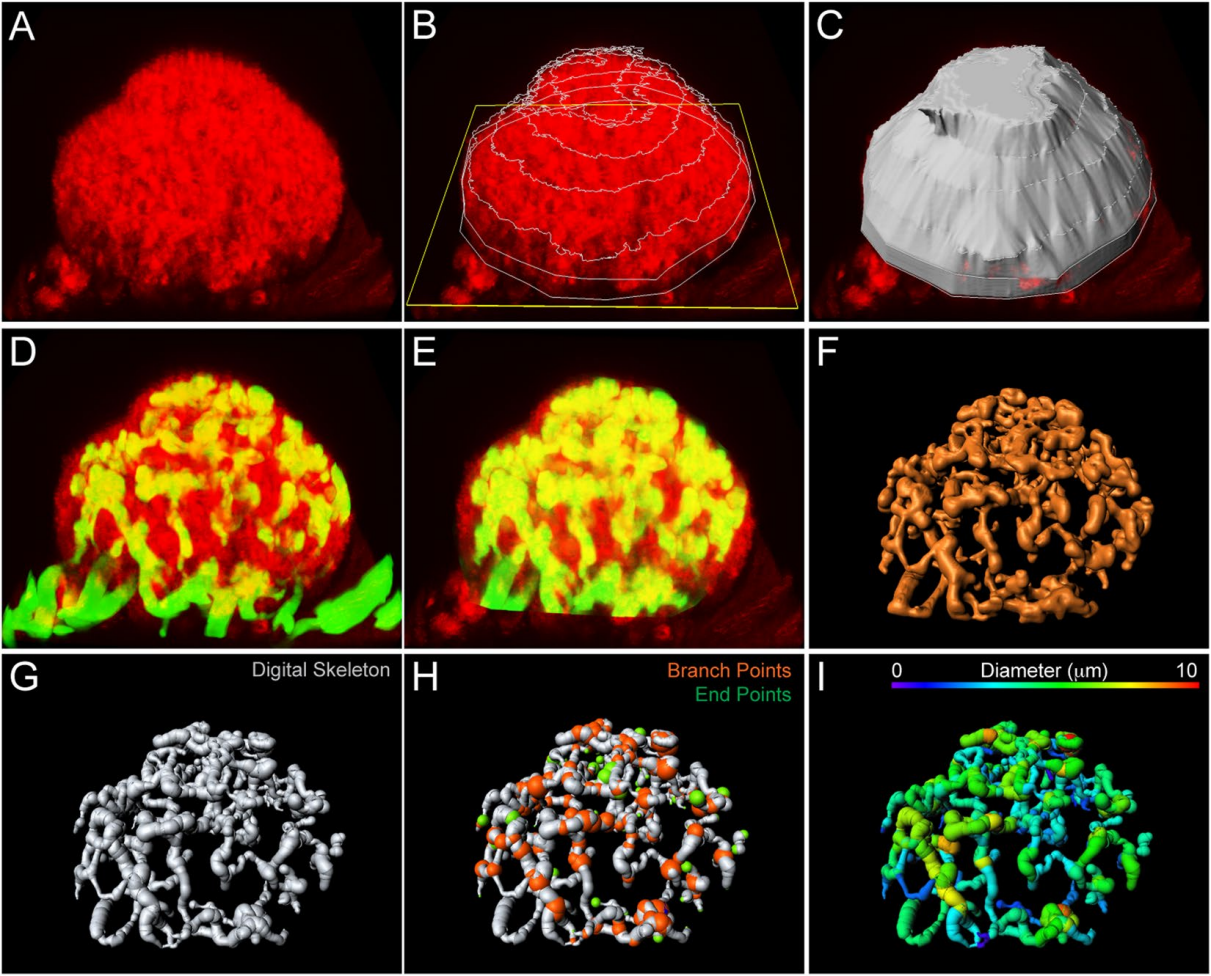
3D quantification of tumour volume and tumour vascularization. (**A**–**C**) Quantification of the tumour volume. The 3D projection of the scattered light (red) collected from the RT2 tumour (**A**) was used to define the tumour surface and base area (**D**–**I**) Multi-step process of quantification of the tumour vasculature. The 3D tumour projection was used to define the tumour vasculature (green) (**D**) in order to remove the iris vasculature (**E**). The fluorescent signal was thresholded to obtain a binary 3D image (**F**), used to create a digital skeleton representing the vasculature (**G**). The skeleton facilitated quantification of various properties such as Branch and End Points (**H**), segment diameter, length and number (**I**).

Quantitative assessment of tumour progression is required to permit evaluation of treatment protocols. To monitor tumour growth we imaged the scattered light from tumour cells by confocal imaging, an approach that requires no cell labelling (Fig. 4A). By rendering a 3D surface projection from the scattered light signal and using the iris plane as the base (Fig. 4B), the tumour volume could be readily quantified (Fig. 4C).

We further developed a multi-step protocol to facilitate quantitative assessment of the complex and irregular morphology of the RT2 tumour vasculature. The tumour vasculature was imaged by TPLSM following administration of FITC-dextran 500 kDa to the circulation (Fig. 4D). The 3D tumour projection was used to define the tumour and to separate the tumour vessels apart from the iris vessels (Fig. 4E). The FITC fluorescence was converted to a binary signal (Fig. 4F), which served as a template to create a digital skeleton representing the vasculature (Fig. 4G). The skeleton facilitates extraction of numerous vascular properties, such as Branch and End Points (Fig. 4H), segment length, number and diameter (Fig. 4I). The multi-step protocol for 3D quantification of tumour growth and tumour angiogenesis is also visualized in the online supplementary material (Supplement Movie 4). Together, the ability to quantify both tumour growth and vascularization in 3D with high precision enables assessment of both tumour progression and treatment efficiency.

### Sunitinib treatment results in tumour vessel regression and reduced RT2 tumour growth

**Figure 5:**
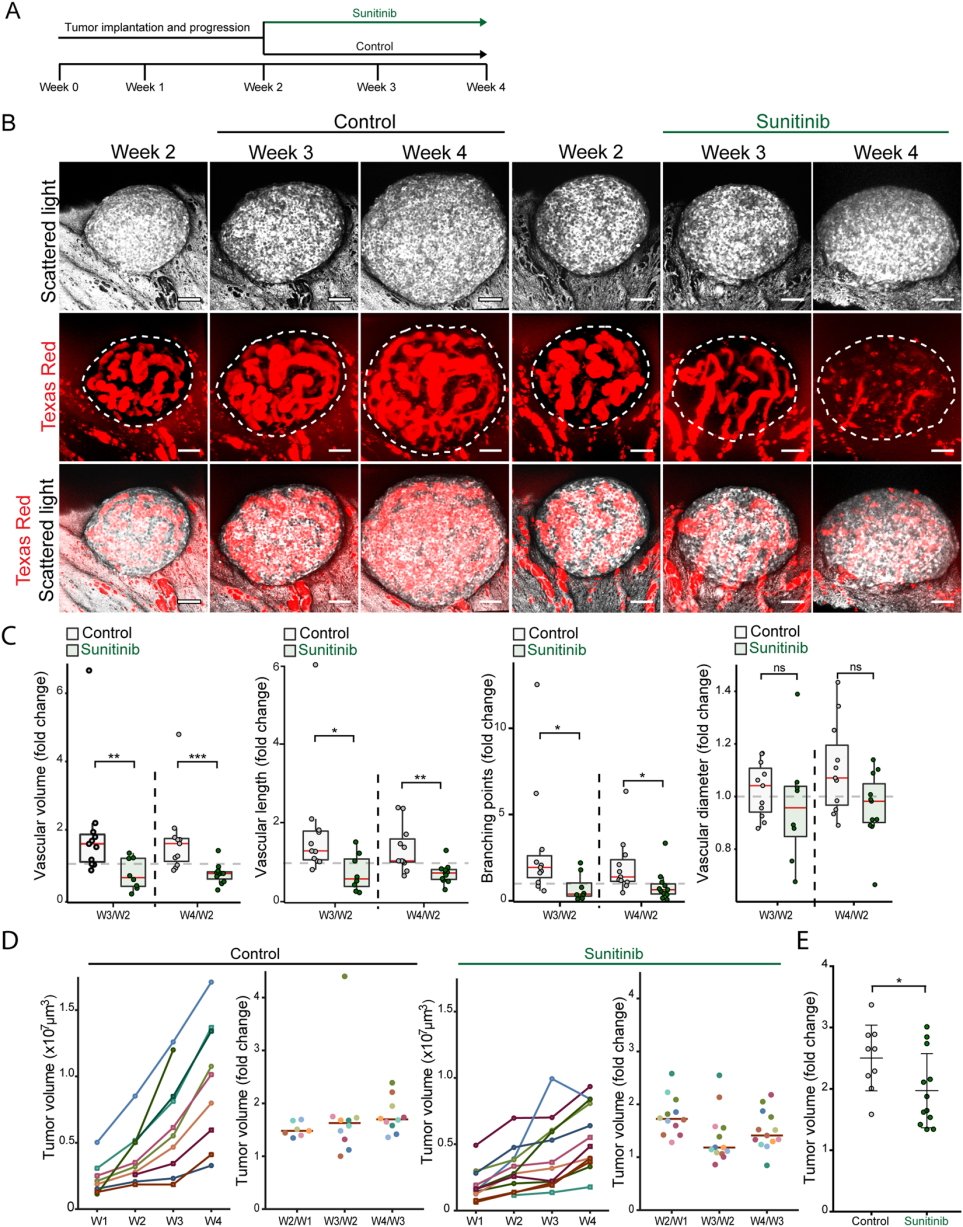
Sunitinib treatment results in tumour vessel regression and reduced RT2 tumour growth. (**A**) Treatment and imaging protocol. (**B**) Image panels showing representative RT2 tumours exposed to sham and Sunitinib treatment at indicated time-points. Tumour cells were visualized by scattered light (grey scale), and tumour vessels by Texas Red-70 kDa dextran administered to the circulation (Texas Red) (red). Bottom row represents merged images. Scale bars represents 50 μm. (**C**) Quantification of indicated tumour vascular parameters showing fold changes after one week (W3/W2) and two weeks (W4/W2) of control and Sunitinib treatment. (**D**) Graphs showing the growth of tumours during the entire study protocol. (E) Fold changes in tumour volume after two weeks of control and Sunitinib treatment. (control = 9, Sunitinib = 12). Statistics: (**C**) Mann-Whitney-Wilcoxon Test, median ± SD, (**E**) Student’s t-test mean ± SD; *P < 0.05, **P < 0.01,***P < 0.001.

Next, we evaluated if our new platform could be applied to investigate drug treatment of RT2 tumours. Pre-oncogenic RT2 islets were implanted into the ACE and allowed to progress for two weeks before the tumour bearing mice were randomly selected for treatment with Sunitinib (40 mg**∕**kg, daily), or control treatment with vehicle (Fig. 5A). Sunitinib resulted in strong regression of tumour vessels already after the first week of treatment (Week 3), which continued during the second week (Week 4) (Fig. 5B). 3D quantification of the tumour vasculature confirmed a significant decrease in the total vascular volume, the total vascular length and in the number of branching points for the Sunitinib treated group, compared to the control treated group (Fig. 5C). There was also a tendency that the tumour vessels became narrower with Sunitinib treatment (Fig. 5C). RT2 tumours exposed to control treatment showed continuous growth during the entire study period (Fig. 5B,D). In contrast, the tumours exposed to Sunitinib treatment almost halted in growth after the first treatment week (Fig. 5D), whereas they resumed growth during the second week, although at a lower rate than prior to the onset of treatment (Fig. 5D). When analysed over the two-week treatment period, Sunitinib resulted in a significant decreased tumour growth rate compared to control treatment (Fig. 5E). Altogether, these data demonstrate the feasibility of monitoring pharmacological efficacy of PNET treatment by intravital microscopy of tumours in the ACE.

### VEGF-B overexpression reduces tumour growth but increases tumour angiogenesis

VEGF-B, originally identified as an endothelial cell growth factor^22^, has been found to play an important role in metabolism by regulating the uptake of fatty acids over the endothelium^23,24^. Additionally, overexpression of VEGF-B was also found to reduce the tumour volume in RT2 transgenes, while genetic depletion of VEGF-B inversely resulted in increased tumour volume^25^. Under both conditions, histological assessment revealed no major affect on tumour vascularization with the exception of altered capillary diameter^25^. To further investigate the effect of VEGF-B overexpression on RT2 tumour progression and tumour angiogenesis, we generated double transgenic RT2-VEGF-B mice, by crossing RT2 mice with transgenic RIP-VEGF-B mice^25^, the latter overexpressing the human form of VEGF-B_167_ under the rat insulin promoter.

**Figure 6:**
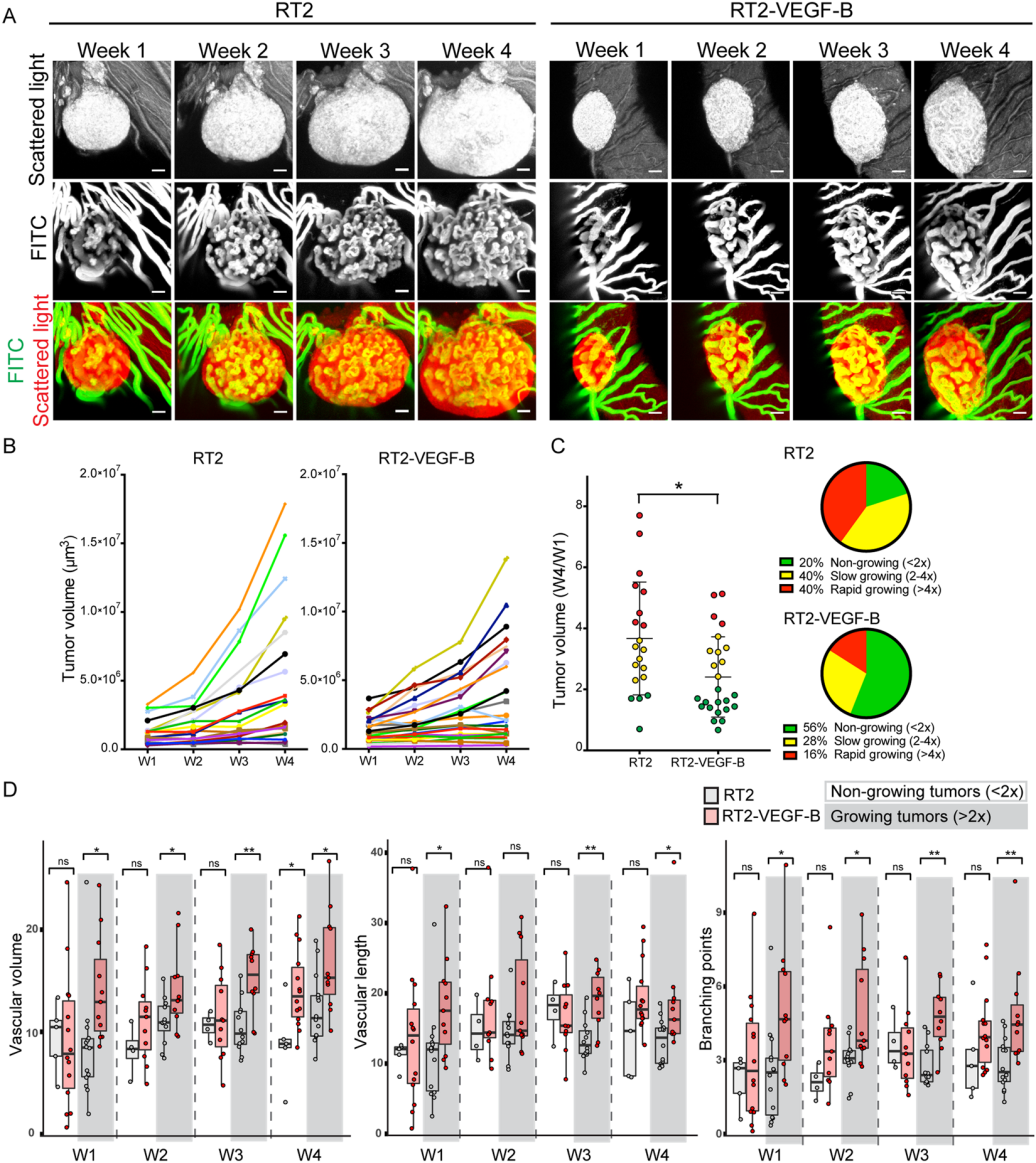
VEGF-B overexpression reduces tumour growth but increases tumour angiogenesis. (**A**) Image panels showing representative RT2 and RT2-VEGF-B tumours at indicated time-points. Tumour cells were visualized by scattered light (grey scale) and tumour vessels by FITC-500 kDa dextran administered to the circulation (FITC) (grey scale). Bottom row represents merged images. Scale bars represent 50 μm. (**B**) Graphs showing the growth of individual tumours during the study. (**C**) Fold changes in tumour volume during the study. The population of non-growing tumours (<2 fold growth) was 20% in the RT2 group and increased to 58% in the RT2-VEGF-B group (RT2 = 20, RT-VEGF-B = 25). (**D**) Quantification of indicated tumour vascular parameters stratified into non-growing tumours (<2 fold growth) and growing tumours (>2 fold) (RT2 = 20, RT-VEGF-B = 25). Statistics: (**C**) Student’s t-test mean ± SD, (**D**) Mann-Whitney-Wilcoxon Test, median ± SD; *P < 0.05, **P < 0.01.

Pre-oncogenic RT2 and RT2-VEGF-B islets were implanted into the ACE of host mice and tumour progression was monitored by intravital microscopy during four weeks. Both RT2 and RT2-VEGF-B tumours engrafted and recruited blood vessels following implantation into the ACE. Notably, VEGF-B overexpression decreased tumour expansion whereas angiogenesis appeared abundant (Fig. 6A). Detailed analysis of individual tumours revealed that a large proportion of the RT2-VEGF-B tumours, 56%, did not grow and displayed <2-fold growth after 4 weeks (Fig. 6B,C). In contrast, only 16% of the RT2 tumours did grow <2-fold during the same period (Fig. 6B,C). Additionally, the number of tumours with >4-fold growth was also reduced in the RT2-VEGF-B group (Fig. 6C). Together, this resulted in 34% reduction of the average tumour volume increase in the RT2-VEGF-B group after 4 weeks of tumour progression (Fig. 6B,C).

Next, we analysed the correlation between the tumour growth rate and the degree of vascularization. The tumours were stratified into two groups with aspect to their growth rate; <2-fold or >2-fold growth. For tumours that did grow <2-fold, the degree of vascularization was similar between the two study groups all 4 time points, with the exception that the RT2-VEGF-B tumours had an increased vascular volume at week 4 (Fig. 6D). For tumours with >2-fold growth, the RT2-VEGF-B group showed increased vascularization at all time points with significantly increased vascular volume, vascular length and number of branch points, compared to the RT2 group (Fig. 6D) However, no difference in vessel diameter was detected (Supplemental Fig. 3). To further investigate the effect of VEGF-B overexpression we also implanted RIP-VEGF-B pancreatic islets into the ACE. Notably, similar to the tumours, normal pancreatic islets with VEGF-B overexpression obtained increased vascular volume and branching points compared to wt islets (Supplement Fig. 4). Additionally, the capillaries in RIP-VEGF-B pancreatic islets acquired a wider diameter compared with capillaries in wt islets, in line with previously observations^25^.

Summarized, our data show that overexpression of VEGF-B suppressed tumour growth and resulted in a growth blockade in a large number of the tumours. Interestingly, VEGF-B overexpression also caused increased angiogenesis both in RT2 tumours and in pancreatic islets.

## Discussion

Here, we have developed a novel model for intravital high-resolution imaging of PNET using confocal and TPLSM imaging. Non-invasive imaging of RT2 tumours in the ACE provides a new platform for 3D visualization and dynamic investigations of RT2 tumour progression and cancer drug efficacy.

The RT2 model has provided valuable knowledge about various aspects of tumour progression, tumour angiogenesis and the tumour microenvironment^2,5,19^. The *in situ* tumour progression has been extensively studied and is characterized by a stepwise development starting around the age of 6 weeks^15^. Isolation of pancreatic islets from 6-week-old RT2 mice yielded islets with a similar size as from non-transgenic wild type mice. RT2 islets that were implanted into the ACE engrafted on the iris and rapidly recruited blood vessels, similar to normal pancreatic islets. Notably, the development of RT2 tumours in the ACE displayed strong similarities to the *in situ* progression in the pancreas. In the pancreas around 50% of the pancreatic islets retain their original size, despite the fact that all beta cells express the SV40T virus^15^. After implantation into the ACE, only 16% of the RT2 tumours retained their size after 4 weeks, indicating favourable conditions for tumour progression. RT2 tumours implanted in the ACE exhibited variable growth rates with volume increases ranging between 2–8-fold after 4 weeks, similar to the heterogeneous size expansion observed in the pancreas^15^. During *in situ* development of RT2 tumours the formation of intratumoural cavities filled with red blood cells, termed “blood islands”, is a hallmark during cancer progression^18,19^. Remarkably, we could also observe the formation of intratumoural cavities in RT2 tumours growing in the ACE. Summarized, these observations show that implantation RT2 tumours into the ACE represent a valid approach to study RT2 tumour growth and progression.

Intravital confocal and TPLSM microscopy provide advantages of high-resolution imaging and 3D visualization, however, a limitation with optical imaging of all tissues is the relatively shallow depth of penetration. By taking advantage of the fact that the RT2 tumours engraft and expand on top of the iris, the tumour volume could be quantified by rendering a 3D surface projection with the iris as a base. Thus, the limitation of tissue penetration could be avoided and the expanding RT2 tumours could readily be imaged. The RT2 tumour vasculature is characterized by a complex and irregular morphology challenging to describe by histological analysis. By applying high-resolution TPLSM and performing 3D reconstruction, together with the application of available image processing tools, our approach facilitates detailed visualization and quantification of the tumour vasculature.

Although tumours in genetically engineered mouse models may not reflect the full spectrum of heterogeneity and diversity seen in human tumours, they are likely to be well suited to evaluate drugs that target the tumour microenvironment as critical signalling axes between cancer cells and the stromal tissue often is preserved between different species^1^. The application of genetic encoded fluorescent reporters offers great possibilities to selectively study distinct tumour and stromal cell populations. Here, the use of Tie2-EYFP mice as tumour hosts, provided visualization of the tumour endothelium in great detail, and help to discriminate between blood vessels and tissue cavities. Additionally, fluorescent labelling of RT2 tumours facilitated identification and monitoring of single tumour cell migration.

Preclinical studies in mice represents an important step in drug development and have contributed to existing PNET therapies^2^. To validate that RT2 tumour progression in the ACE represent a valuable platform for cancer drug assessment, we evaluated the affect of Sunitinib treatment. Sunitinib, a multi-tyrosine kinase inhibitor, is a proven angiogenesis inhibitor shown to impair tumour burden, vessel density and blood flow in previous pre-clinical studies^26,27^, and is currently approved for PNET therapy. Sunitinib treatment of established tumours caused significant vessel regression after one week of treatment, which persisted during the second week of treatment. Vessel regression was paralleled by impaired tumour growth, which was strongest during the first week of treatment, and significant after 2 weeks. Notably, a trend for tumour growth recovery could be observed during the second week of treatment, which previously has been observed with longer periods of VEGF inhibition^19^. These data clearly show that intravital imaging of RT2 tumours in the ACE represent a valid platform for evaluation of cancer drug efficacy for PNET therapy.

Next, we applied this new platform to investigate the affect of VEGF-B overexpression on RT2 tumour progression. Repetitive imaging of individual tumours revealed that VEGF-B overexpression suppressed the tumour expansion. Remarkably, a large fraction of the VEGF-B overexpressing tumours, 56% compared to 16% in the control group, did not grow but retained their initial size during the study period, suggesting that VEGF-B overexpression repress oncogenic transformation. Our results are in line with previous studies showing that overexpression of VEGF-B suppress the growth of RT2 tumours^25^ and human melanoma cells^28^. Interestingly, VEGF-B overexpression resulted in a similar or increased degree of vascularization, both at early and late time-points of tumour progression. This indicates that VEGF-B overexpression contributes to an inverse correlation between tumour growth and vascularization. Moreover, VEGF-B overexpression also resulted in increased revascularization of pancreatic islets, indicating that VEGF-B overexpression stimulates angiogenesis in both normal and oncogenic pancreatic tissue. Further studies are required to disclose the mechanisms underlying these observations. Interestingly, VEGF-B play an important part in the metabolism of healthy tissue by regulating the transport of long-chain fatty acids over the endothelium^23^. Thus, it could thus be speculated that VEGF-B also affect tumour metabolism. Genetic deletion of VEGF-B caused reduced lipid uptake to heart tissue, while glucose uptake was concomitantly increased^23^. Since, depletion of VEGF-B correlates with increased tumour growth^25^, it could be speculated that a metabolic shift with increased access to glucose fuels tumour progression in this context. Along the same line, a metabolic shift diminishing glucose availability due to VEGF-B overexpression, could thus suggestively contribute to suppressed tumour growth.

In addition to its role in metabolism, VEGF-B is also part of the VEGF family and binds to the VEGFR-1 in competition with VEGF-A. VEGFR-1 is recognized as a decoy receptor for VEGF-A, which sequesters VEGF-A and reduces its binding of to VEGFR-2^29^. Thus, VEGF-B excess due to overexpression could outcompete VEGF-A from VEGFR-1, and thereby result in increased VEGF-A/VEGFR-2 signalling and enhanced tumour angiogenesis, as has been demonstrated in adipose tissue^30^.

Noninvasive intravital imaging in the ACE has successfully been applied to study multiple aspects of beta cell physiology^31^, as well as pancreatic development^32^ and glomeruli function^33^. Moreover, successful application of additional high-resolution imaging techniques, such as optical coherence tomography (OCT)^34^, further support the application of the ACE for intravital imaging. Here, we present a novel platform for noninvasive intravital high-resolution imaging of PNET progression and cancer drug efficacy. This platform opens up a new window of opportunities to study complex cellular processes in tumour progression and treatment strategies by longitudinal and high-resolution live imaging.

## Materials and Methods

### Animals

All animal procedures were performed in accordance with relevant guidelines and regulations and approved by the Stockholm North Ethical Committee on Animal Research, Stockholm, Sweden. The following mouse strains were used: RIP1-Tag2 (RT2)^3^, Tie2-Cre^tg/wt16^, R26R-Ai3-EYFP^fl/wt17^, RIP-Cre^Tg/Wt21^, R26R-Ai14-Tomato^fl/wt17^, RIP-VEGF-B^25^ and maintained on C57Bl/6 J background.

For Sunitinib treatment, Sunitinib (SelleckChem) was dissolved at 80 mg/ml in 100% DMSO (Sigma), allocated and frozen at −21 °C for up to 1 week. Pure DMSO was similarly allocated and frozen. 200 μl DMSO, with Sunitinib or without (vehicle), were freshly mixed with 400 μl PEG400 (Sigma) and 400 μl DPBS (Sigma) and injected subcutaneous within 30 min. Every mouse received 50 μl/day for 14 consecutive days of the Sunitinib solution (treatment) or vehicle (sham treatment).

### Islet isolation and transplantation to the anterior chamber of the eye

Islet isolation was performed as described^14^. Briefly, the pancreas was filled with 1 mg/mL collagenase (Collagenase A; Roche) and kept on ice until digestion at 37 °C, between 8–12 min. The digestion was stopped with ice-cold HBSS supplemented with 1% BSA/25 mM Hepes, and the islets were purified by hand picking. Implantation of islets into the anterior chamber of the eye (ACE) was conducted as described in detail^12^.

### Intravital imaging

Intravital imaging of islets in the ACE was conducted as described in detail^12^. Briefly, the mice were anesthetized using 2% isoflurane anaesthesia and injected with the indicated vascular tracer and placed in a custom designed head holder that to allow stabilization of the eye. Imaging was performed using an upright Leica TCS SP8 MP microscope, equipped with a 25×/1 NA long working distance immersion lens (Leica, 507704). Two-photon microscopy was conducted with external Coherent Cameleon VisionS laser and HyD or PMT detectors. Fluorophores were excited at 820 nm or 960 nm. Fluorescence was collected using the following filter cubes: 447/60 and 525/50 (blue/green), 607/70 and 697/75 (orange/red) with a LP560 main beam splitter, and. Scattered light, i.e. non-fluorescent light dispersed by the densely granule packed hormone producing cells, was collected by confocal imaging of as described^12^. Images were registered in bidirectional non-resonant scanning mode with a step size of 1 μm for stacks, using the minimal necessary laser power (optical power meter, Thorlabs, PM100D). Images were collected in 8-bit format and pseudo coloured as indicated with black corresponding to the lowest intensity level. All images are maximum projections of image Z-stacks and are displayed close to the original intensity. For recording of Z-stacks, the signal in the upper image planes was set close to saturation, while the signal gradually became weaker with increased imaging depth, as illustrated in Supplement Movie 2 and 3.

### 3D quantification of the tumour volume and the tumour vasculature

Image processing (denoising, enhancing signal intensity) and visualization was performed with Imaris Software (Bitplane) and Fiji/ImageJ (NIH). Each individual image was registered if necessary for correction of mouse movement during the image acquisition and filtered by Median 3D filter (Fiji). Manual 3D cropping to remove the iris vasculature was performed with use of Fiji Segmentation Editor in user developed macro. A binary 3D image of blood vessels was obtained by thresholding the image previously enhanced by Tubeness plugin^35^. Skeletonization and manual correction was performed in Imaris software (Bitplane), and was followed by extraction of parameters of 3D vascular network. Definition of vascular parameters; Vascular length: total length of vasculature per (10000 µm³) of islet volume; Vascular volume: total volume of vasculature per (%) of islet volume; Branching points: total number of branching points per (100000 µm³ = 0.0001 mm³) of islet volume; Mean Diameter: weighted arithmetic mean of filaments diameter weighted by the length of filaments.

### Statistical analysis

Statistical analysis was performed with the GraphPad Prism 6.0 (GraphPad Software, Inc., La Jolla, CA, USA). All tests of statistical significance were two-sided. Unpaired Student’s t-test was used to study differences between two groups. In cases of unequal variance, Welch’s t-test was used. *p* values below 0.05 were considered significant. In all figures, data points are presented on scatter plots with means +/− SD.

## Supplementary information

Supplement Information

Supplement Movie 1

Supplement Movie 2

Supplement Movie 3

Supplement Movie 4

## Data Availability

The datasets generated during and/or analysed during the current study are available from the corresponding author on reasonable request.
