# Supplementary material for: Noninvasive intravital high-resolution imaging of pancreatic neuroendocrine tumours
Source: Sci Rep. 2019 Oct 10;9:14636. doi: 10.1038/s41598-019-51093-0 (PMC6787246; doi:10.1038/s41598-019-51093-0)

Noninvasive intravital high-resolution imaging of pancreatic neuroendocrine tumours

Mirela Balan[1], Marta Trusohamn[1], Frank Chenfei Ning[1], Stefan Jacob[2], Kristian Pietras[3], Ulf Eriksson[1], Per-Olof Berggren[2], Daniel Nyqvist[1*]

SUPPLEMENTARY DATA

Supplemental Figure 1. (A) Overview images captured with a stereomicroscope showing one eye after implantation of 2 RT2 tumour islets (arrows) and 2 normal pancreatic islets (arrowheads) at the indicated time-points. (B) Image panel (maximum projections) of a normal pancreatic islet imaged at indicated time-points following implantation. The islet cells were imaged by detection of scattered light, and the islet vasculature were imaged by systemic administration of FITC-dextran 500 kDA captured by either confocal (1P) or TPSLM imaging (2P). Bottom row represents the merged scattered light (grey scale) and FITC-1P (green).

Supplemental Figure 2. Confocal images show a cryosection of a RT2-Tomato tumour fixed four weeks after implantation and stained for DAPI (blue) and insulin (grayscale). The section confirms that the RT2 tumours grow on top of the iris. Moreover, a strong overlap between the insulin staining and tdTomato fluorescence (red) can be observed. (n=3) Scale bar = 50 μm.

Supplemental Figure 3. Quantification of the mean vessel diameter stratified into non-growing tumours (<2 fold growth) and growing tumours (> 2 fold) (RT2=20, RT-VEGF-B=25). Statistics: Mann-Whitney-Wilcoxon Test, median±SD; ns= non-significant

Supplemental Figure 4. VEGF-B overexpression increases the vessel diameter in revascularized pancreatic islets. (A) Image panels showing representative wildtype (wt) and RIP-VEGF-B pancreatic islets at indicated time-points. Islet cells were visualized by scattered light and tumour vessels by Texas Red-70 kDa dextran administered to the circulation (Texas Red). Bottom row represents merged images. Scale bars represent 50 μm. (B) Quantification of indicated islet vascular parameters (wt>15, RIP-VEGF-B>15). Statistics: (Mann-Whitney-Wilcoxon Test, median±SD; *P< 0.05, **P< 0.01, ns= non-significant.

Supplemental Movie 1
The movie captured with wide-field microscopy shows a RT2 tumour and real-time blood flow after intravenous injection of FITC-500kDa. The arrow indicates the flow direction in a tumour microvessel that exhibits alternating flow directions.

Supplemental Movie 2
The movie shows a confocal image stack of a RT2 tumour. The left image shows the Scatter light from the tumour cells (grayscale) and the right image shows intravenous injected Texas Red-70kDa (red). Intratumoural cavities can be observed as black spaces in the scattered light images, and to be filled with Texas Red dye.

Supplemental Movie 3

The movie shows the image Z-stack of RT2 tumour displayed in Figure 3 at Week 4. The left image shows the Scatter light from the tumour cells (grayscale) and the right image shows the tdTomato fluorescence from the tumour cells (grayscale). Higher cellular detail and greater imaging depth was obtained with the tdTomato reporter. The number in the top left corner indicates the image plane (step size = 1μm).

Supplemental Movie 4. The movie illustrates the multi-step protocol for 3D quantification of tumour growth and tumour angiogenesis.

Supplemental Figure 1

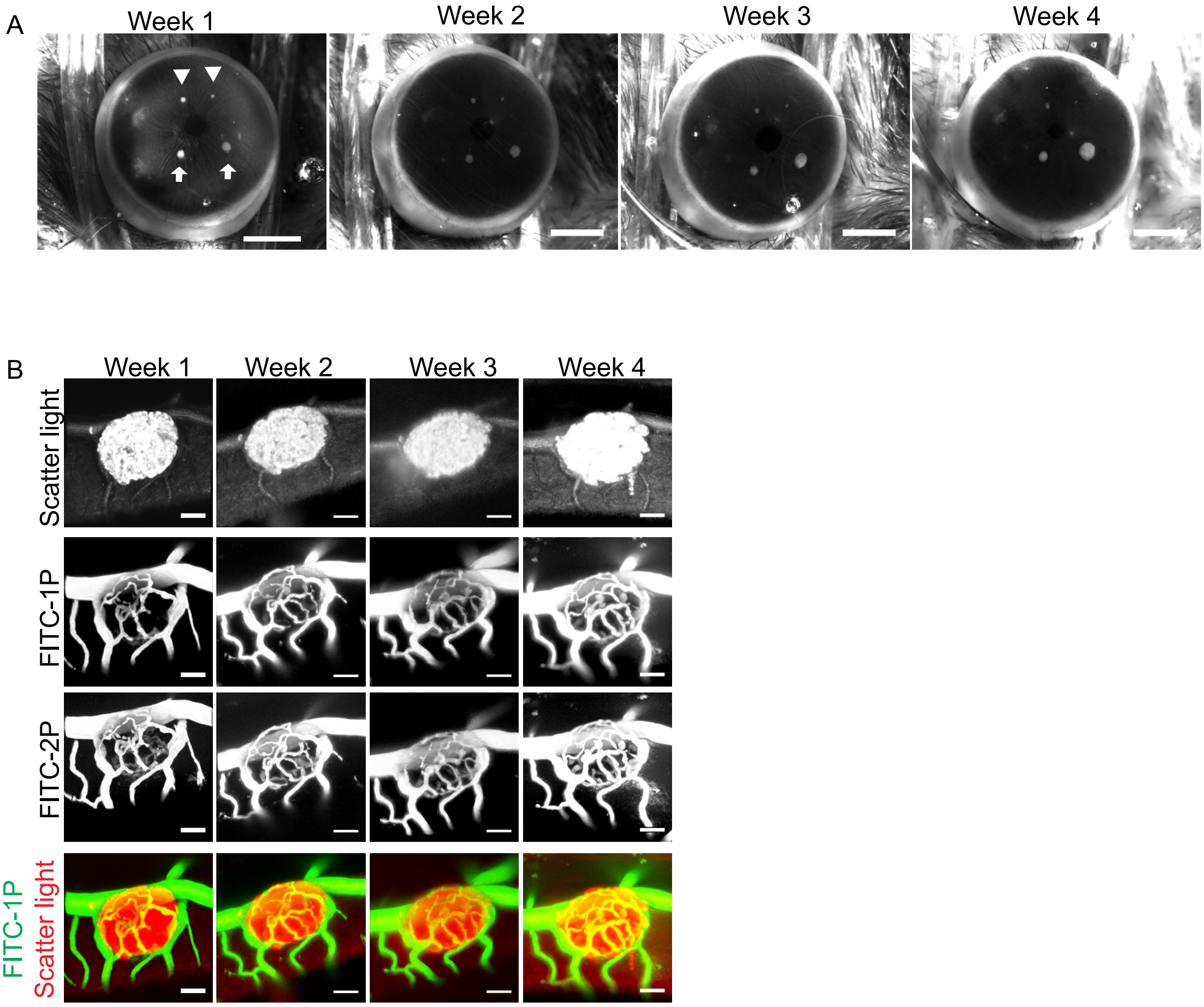

Supplemental Figure 2

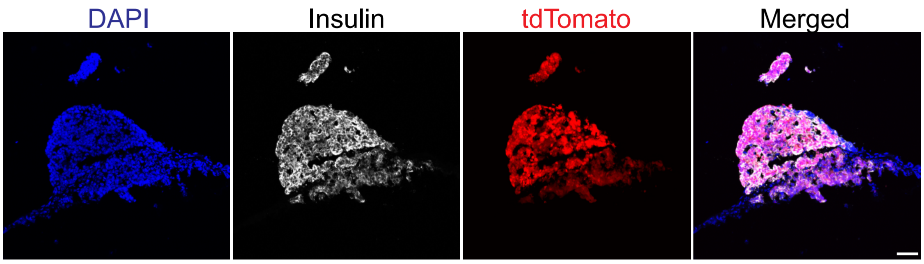

Supplemental Figure 3

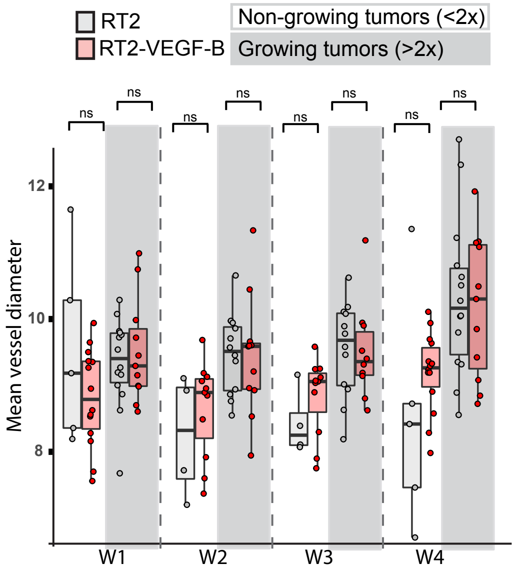

Supplemental Figure 4

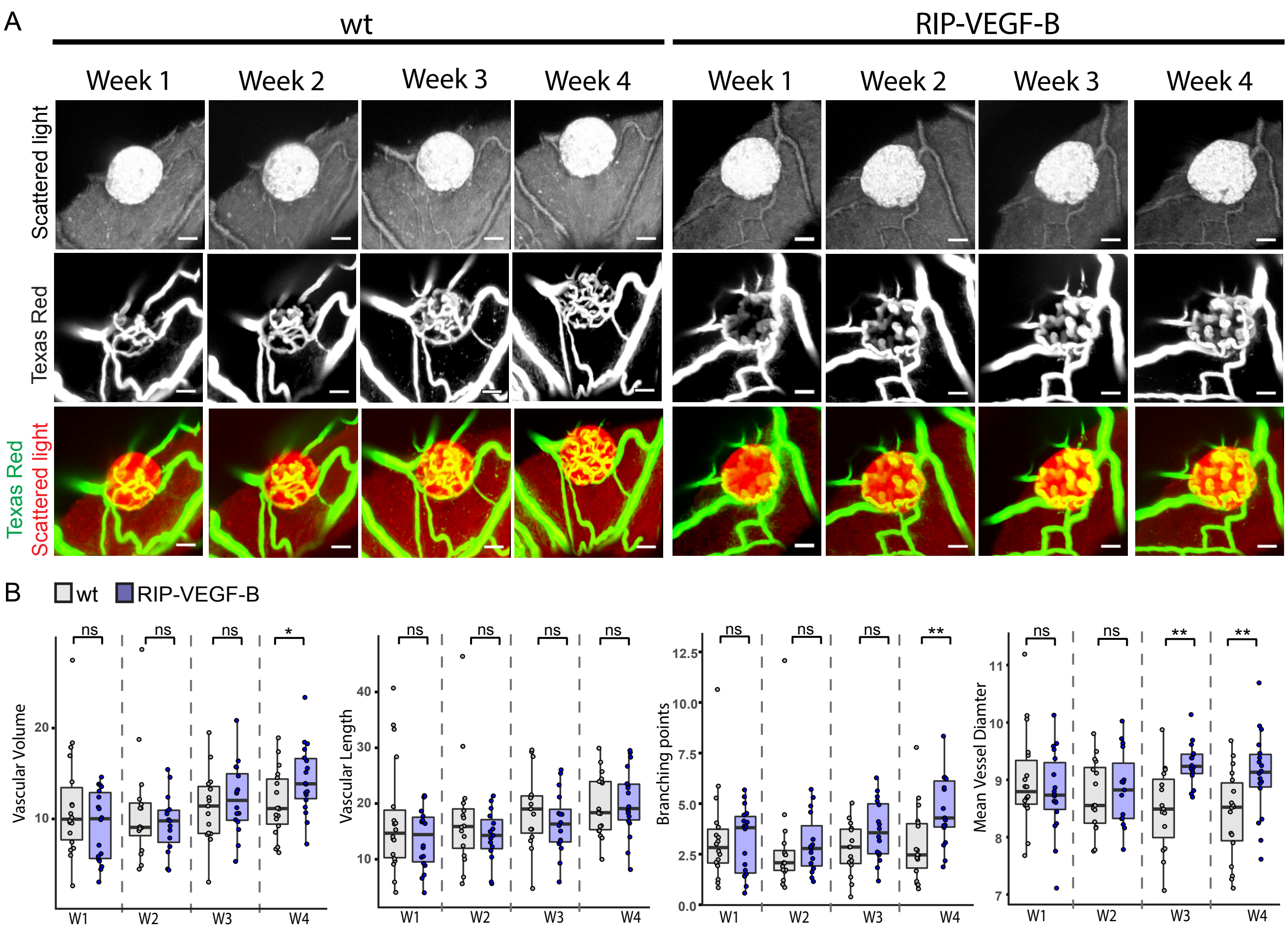

Supplement: Supplementary file 1 — Supplement Information [file 41598_2019_51093_MOESM1_ESM.pdf]
